# The trefoil factor interacting protein TFIZ1 binds the trefoil protein TFF1 preferentially in normal gastric mucosal cells but the co-expression of these proteins is deregulated in gastric cancer

**DOI:** 10.1016/j.biocel.2008.07.015

**Published:** 2009-03

**Authors:** Felicity E.B. May, S. Michael Griffin, Bruce R. Westley

**Affiliations:** a Department of Pathology, University of Newcastle upon Tyne, Royal Victoria Infirmary, Queen Victoria Road, Newcastle upon Tyne NE1 4LP, UK; b Northern Institute for Cancer Research, School of Clinical and Laboratory Sciences, The Medical School, University of Newcastle upon Tyne, Newcastle upon Tyne NE2 4HH, UK; c Northern Oesophago Gastric Cancer Unit, Royal Victoria Infirmary, Queen Victoria Road, Newcastle upon Tyne NE1 4LP, UK

**Keywords:** TFIZ1, Trefoil proteins, Gastric mucosa, Gastric cancer, Metastasis

## Abstract

The gastric tumour suppressor trefoil protein TFF1 is present as a covalently bound heterodimer with a previously uncharacterised protein, TFIZ1, in normal human gastric mucosa. The purpose of this research was firstly to examine the molecular forms of TFIZ1 present, secondly to determine if TFIZ1 binds other proteins apart form TFF1 *in vivo*, thirdly to investigate if TFIZ1 and TFF1 are co-regulated in normal gastric mucosa and fourthly to determine if their co-regulation is maintained or disrupted in gastric cancer. We demonstrate that almost all human TFIZ1 is present as a heterodimer with TFF1 and that TFIZ1 is not bound to either of the other two trefoil proteins, TFF2 and TFF3. TFIZ1 and TFF1 are co-expressed by the surface mucus secretory cells throughout the stomach and the molecular forms of each protein are affected by the relative abundance of the other. TFIZ1 expression is lost consistently, early and permanently in gastric tumour cells. In contrast, TFF1 is sometimes expressed in the absence of TFIZ1 in gastric cancer cells and this expression is associated with metastasis (lymph node involvement: *p* = 0.007). In conclusion, formation of the heterodimer between TFIZ1 and TFF1 is a specific interaction that occurs uniquely in the mucus secretory cells of the stomach, co-expression of the two proteins is disrupted in gastric cancer and expression of TFF1 in the absence of TFIZ1 is associated with a more invasive and metastatic phenotype. This indicates that TFF1 expression in the absence of TFIZ1 expression has potentially deleterious consequences in gastric cancer.

## 1 Introduction

Our interest in the trefoil factor interactions(z) 1 (TFIZ1) protein stems from the finding that it forms a heterodimer with the trefoil protein TFF1 (Westley et al., 2005). The protein was identified independently using two other approaches. It was described in the Chinese literature as a gene whose expression is decreased in gastric cancer (Du et al., 2003) and as a gene whose expression is increased following *Helicobacter pylori* eradication (Resnick et al., 2006). TFIZ1 is an 18.3 kDa secreted protein (Westley et al., 2005) encoded by one of two closely related genes on chromosome 2 (2p14), also known as GDDR (down-regulated in gastric cancer) (Du et al., 2003, 2005), blottin (Otto et al., 2006) and gastrokine 2 (GKN2) (Baus-Loncar et al., 2007).

TFIZ1 is related to GKN1 which is also known as CA11 (Yoshikawa et al., 2000), AMP-18 (Martin et al., 2003), foveolin (Oien et al., 2004) and TFIZ2 (Westley et al., 2005). Similarly to TFIZ1, the expression of one isoform of GKN1 is also decreased by *H. pylori* (Nardone et al., 2007; Resnick et al., 2006). The functions of TFIZ1 and GKN1 are not known, but it has been suggested that they are involved in the protection of the gastric mucosa and it has been reported that peptide fragments of GKN1 are both mitogenic and motogenic (Toback et al., 2003). TFIZ1 and GKN1 both contain a Brichos domain (Sanchez-Pulido et al., 2002). This domain is approximately 100 amino acids long and it is found in a variety of proteins implicated in dementia, respiratory distress and cancer.

TFF1 is a small (6.67 kDa) secreted protein identified originally as an oestrogen-regulated mRNA in breast cancer cells (Masiakowski et al., 1982; May and Westley, 1986; Ribieras et al., 1998). It is one of three trefoil proteins all of which contain one or two copies of the 42–43 amino acid trefoil domain that has six conserved cysteine residues (Ribieras et al., 1998; May and Westley, 1997). TFF1 and TFF3 contain one trefoil domain while TFF2 contains two domains.

Trefoil proteins are synthesized by mucus secreting epithelia and are important in mucosal protection. The main site of expression of TFF1 in normal tissues is the stomach (Rio et al., 1988; Piggott et al., 1991; Madsen et al., 2007). TFF1 has been shown to interact directly with mucins both *in vitro* and *in vivo* (Tomasetto et al., 2000; Ruchaud-Sparagano et al., 2004). TFF1 protects from experimentally induced gastrointestinal damage and is thought to promote restitution (Playford et al., 1996; Marchbank et al., 1998). Its interaction with human MUC5AC (Ruchaud-Sparagano et al., 2004), which is proposed to strengthen the adherent mucus gel barrier (Thim et al., 2002), is exploited by the Class I carcinogen *H. pylori* (Clyne et al., 2004).

**Fig. 1:**
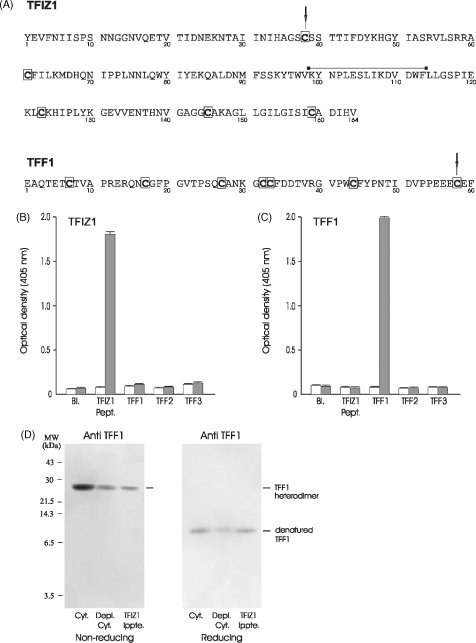
Co-immunoprecipitation of TFF1 with TFIZ1 antiserum. The amino acid sequences of mature TFIZ1 and TFF1 are shown (A) with the cysteine residues involved in the intermolecular disulphide bond indicated by downward pointing arrows. Cysteine residues are shown in bold and are boxed. Rabbits were immunized with a synthetic 15 residue TFIZ1 peptide predicted to be located in a solvent accessible region of the protein. TFIZ1 peptide, and recombinant TFF1, TFF2 and TFF3 were coated onto ELISA plates in 50 mM sodium bicarbonate pH 9.5. Wells were incubated with pre-immune rabbit serum ((□) B) or TFIZ1 antiserum ((■) B) or with mouse IgG ((□) C) or purified anti TFF1 monoclonal antibody ((■) C) and the amount of antibody bound measured using an alkaline phosphatase-conjugated secondary antibody. TFIZ1 was immunoprecipitated from gastric cytosol. Aliquots of gastric cytosol (Cyt.), immuno-depleted cytosol (Depl. Cyt.) and TFIZ1 immunoprecipitate (TFIZ1 Ippte.) were electrophoresed on polyacrylamide gels without (non-reducing) or after (reducing) prior incubation with β-mercaptoethanol (D). The proteins were transferred to PVDF membrane and incubated with TFF1 antibody. The positions of the molecular mass markers are shown on the left and the forms of TFF1 on the right of the panels.

In contrast to the defined loop structure of the trefoil domain, the carboxy-termini of both TFF1 (Polshakov et al., 1997) and TFF3 (Lemercinier et al., 2001) monomers are unstructured. TFF1 and TFF3 have an extra cysteine residue near the carboxy-terminus that mediates intermolecular interactions (Fig. 1A). TFIZ1 has an odd number of cysteine residues (Westley et al., 2005) and the TFIZ1:TFF1 heterodimer contains an intermolecular disulphide bond formed most probably between Cys38 of TFIZ1 and Cys58 of TFF1 (Fig. 1).

The predominant molecular form of TFF1 identified in human gastric mucosa is the heterodimer of ∼25 kDa with TFIZ1, but it is also present as a monomer and homodimer (Ruchaud-Sparagano et al., 2004; Newton et al., 2000). In TFF1 homodimers, the two monomer units are connected by a flexible linker comprising the two carboxy-termini (Chadwick et al., 1997; Williams et al., 2001). The TFF1 homodimer has greater activity than the monomer *in vitro* and in animal models (Prest et al., 2002; Marchbank et al., 1998). Of the three molecular forms of TFF1 identified, the TFF1 homodimer is bound most strongly to gastric mucins and interacts preferentially with MUC5AC (Ruchaud-Sparagano et al., 2004; Newton et al., 2000). *H. pylori* have been shown to interact with the TFF1 homodimer but not the TFF1 monomer, and it has been proposed that *H. pylori* colonize the adherent mucus layer as a consequence of the specific interaction with the TFF1 homodimer (Clyne et al., 2004).

The identification of TFIZ1 as the partner of TFF1 in the heterodimer raises several interesting biological questions about this protein. In this study, the spatial expression of TFIZ1 and the molecular forms of TFIZ1 in the stomach are defined, and the specificity of the TFIZ1 TFF1 interaction is investigated. The patterns of expression of TFIZ1 and TFF1 in gastric cancer are also examined as this could shed light on the contribution of TFIZ1 to the tumour suppressor activity of TFF1.

## 2 Materials and methods

### 2.1 Collection of gastric samples

Ethical permission for the studies was obtained from the Joint Newcastle Health Hospitals/University of Newcastle upon Tyne Ethical Committee. Informed consent for the use of normal gastric mucosal samples was obtained from participants in this study.

### 2.2 Preparation of cytosol from gastric mucosa

Normal gastric mucosa was taken from gastrectomy specimens resected as primary therapy for gastric cancer. The mucosa was taken from as far as was practicable from the tumour. Cytosol was prepared as described previously (Newton et al., 2000) except that the tissue sample was pulverised after cooling in liquid nitrogen using a micro-dismembrator prior to homogenisation of the resultant powder in inhibitor buffer using an ultra-turrax homogeniser. Each 100 mg of pulverised tissue was suspended in 1 ml of inhibitor buffer: 1 mM iodoacetamide, 4 mM PMSF, 5 mM benzamidine HCl, 10 mM EDTA, 100 mM α-aminocaproic acid, 10 mM *N*-ethyl maleimide, 67 mM sodium phosphate pH 6.5. After centrifugation for 1 h at 100 000 × *g*, the cytosol was stored at −70 °C. The protein concentrations of the cytosols were measured using the bicinchoninic acid protein assay.

### 2.3 Production of recombinant trefoil proteins

Recombinant human TFF1 and TFF3 were produced by periplasmic expression from *E. coli* (May et al., 2003; Chadwick et al., 1997). The TFF1 and TFF3 monomers and dimers were purified by a combination of affinity chromatography, anion exchange chromatography and gel filtration (May et al., 2000). Recombinant human TFF2 was produced by secretion from *P. pastoris*. Non glycosylated and glycosylated TFF2 were purified by cation and anion exchange and affinity chromatography.

### 2.4 Preparation of trefoil factor family protein and TFIZ1 antibodies

Mice were immunized with correctly folded TFF1 or TFF3 dimer or TFF1 or TFF2 peptides. Hybridoma cell lines were produced and screened by ELISA (Westley et al., 2005; Pu et al., 2004). A TFIZ1 peptide of 15 amino acids that was predicted to be immunogenic was synthesized, conjugated to KLH and used to immunize rabbits. Antibody production and titre were monitored by ELISA.

### 2.5 ELISA

Microtitre plate wells were coated with 50 μl of 200 ng/ml peptide or 400 ng/ml trefoil protein and processed essentially as described previously (Pu et al., 2004). Plates were washed and incubated with TFF1 monoclonal antibody hybridoma supernatant or TFIZ1 antisera followed by the appropriate secondary antibody conjugated to alkaline phosphatase. The reaction was visualized with *para*-nitro phenyl phosphate (Pu et al., 2004).

### 2.6 Immunoprecipitation

TFF1 monoclonal antibodies were purified on protein A Sepharose from protein-free hybridoma supernatant and conjugated to protein G Sepharose using disuccinimidyl suberate. TFIZ1 polyclonal antisera were affinity purified on the basis of immunoreactivity with the immunizing peptides prior to conjugation to protein G Sepharose. Cytosols, prepared as described previously (Newton et al., 2000), were preabsorbed with protein G Sepharose and incubated with the conjugated antibodies in 300 mM NaCl, 100 mM Tris–HCl, pH 8.0, 0.2% Nonidet-P40 at 4 °C overnight. The immunoprecipitated proteins were collected by centrifugation, washed 3 times in the above buffer and eluted in non-reducing sample buffer (Pierce).

### 2.7 Protein gel electrophoresis and Western transfer analysis

Proteins were electrophoresed on gradient polyacrylamide gels and transferred to PVDF membranes as described previously (Chadwick et al., 1997). Proteins were boiled for 5 min in the absence or presence of β-mercaptoethanol prior to electrophoresis. Filters were incubated with TFF1, TFF2 or TFF3 monoclonal antibodies (Westley et al., 2005; May et al., 2004) or TFIZ1 rabbit antiserum diluted in 5% milk, followed by alkaline phosphatase-conjugated secondary antibodies and the immunological reaction developed with nitro blue tetrazolium and 5-bromo-4-chloro-3-indolyl phosphate (Chadwick et al., 1995).

### 2.8 Immunohistochemistry

Sections of formalin-fixed, paraffin-embedded gastric mucosa were processed as described previously except that heat retrieval was for 1 or 3.5 min in a pressure cooker in 10 mM sodium citrate, pH 6.0 (Crosier et al., 2001). Endogenous biotin was blocked with the Dako Cytomation blocking system. The sections were incubated with the primary antibodies for 1 h, with the secondary antibody for 30 min and with avidin–biotin immunoperoxidase complex (Vector laboratories) for 30 min at room temperature, developed with diaminobenzidine and counterstained with haematoxylin.

## 3 Results

### 3.1 Preparation and evaluation of TFIZ1 antibodies

The sequence of secreted 18.3 kDa TFIZ1 protein is shown in Fig. 1A. Specific TFIZ1 antisera were raised to enable us to study the molecular forms of TFIZ1 and their expression in normal and malignant gastrointestinal mucosa. The effectiveness and specificity of the TFIZ1 antisera and TFF1 monoclonal antibodies were tested by ELISA (Fig. 1B and C). There was a strong reaction against the TFIZ1 peptide and no reaction against TFF1, TFF2 or TFF3 recombinant protein. The TFF1 monoclonal antibody was specific for TFF1 and did not react with TFIZ1 peptide, TFF2 or TFF3. The TFIZ1 antiserum was tested for its ability to immunoprecipitate the TFF1:TFIZ1 heterodimer from protein extracts of normal human gastric mucosa. Aliquots of human gastric cytosol, of the same cytosol after removal of the proteins that were bound by the TFIZ1 antisera, and of the proteins immunoprecipitated by the TFIZ1 antisera were electrophoresed under non-reducing conditions, transferred to PVDF membrane and incubated with TFF1 antibody (Fig. 1D). The TFF1 heterodimer is present in all three samples which indicates that some but not all of the TFF1:TFIZ1 heterodimer has been immunoprecipitated by the TFIZ1 antisera. Analysis after electrophoresis under reducing conditions with a TFF1 antibody confirms that TFF1 has been co-immunoprecipitated by the TFIZ1 antiserum (Fig. 1D). Control experiments in which protein extracts of normal gastric mucosa were incubated with the pre-immune serum did not co-immunoprecipitate TFF1.

### 3.2 Location of TFIZ1 in the gastric mucosa

**Fig. 2:**
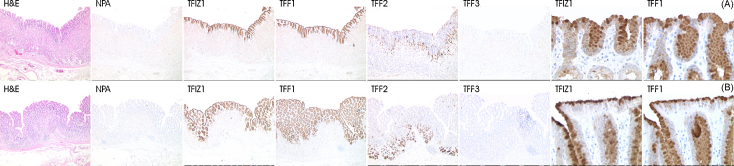
Distribution and co-expression of TFIZ1, TFF1, TFF2 and TFF3 in normal human gastric mucosa. Sections of gastric mucosa body (A) and antrum (B) regions were processed for immunohistochemistry to locate TFIZ1, TFF1, TFF2 and TFF3. Haematoxylin and eosin staining of the full depth of the mucosa and underlying submucosa are shown (H&E). Sections processed for immunohistochemistry in the absence of primary antibody show low diffuse brown background reaction (NPA). Immuno-reaction for TFIZ1 and TFF1 is restricted to the surface and foveolar epithelial mucus secretory cells, TFF2 to cells in the mucus neck and basal portions of the gastric glands. TFF3 was not detected. Higher magnification photomicrographs for TFIZ1 and TFF1 immuno-reaction show the presence of both proteins in the cytoplasm and within the mucus globules in the apical regions of surface cells and cells lining the foveoli. The original magnifications for the photomicrographs shown in the panels on the left hand side of the figure is ×40 and for the panels on the right hand side of the figure is ×400. (For interpretation of the references to color in this figure legend, the reader is referred to the web version of the article.)

The predominant form of TFF1 extracted from the normal gastric mucosa is a heterodimer with TFIZ1. It is likely that the heterodimer is formed intra-cellularly in single cells but it is possible, because an extract would contain proteins secreted from cells at the base of gastric glands and from cells in the foveolae, that it is formed after secretion into the extra cellular environment following the synthesis of the two proteins in separate cell types. In addition, TFIZ1 may be expressed more widely than TFF1 and form heterodimers with other proteins in cells that do not express TFF1. To investigate this, the localisation of TFIZ1 and that of three trefoil proteins was determined by immunohistochemistry in mucosal sections from different regions of the stomach. Typical results obtained with sections of gastric body and antrum are shown in Fig. 2. TFF1 was localised with the monoclonal antibody used previously to purify the heterodimer, TFF2 and TFF3 using monoclonal antibodies, and TFIZ1 with the polyclonal antibody described in Section 2 and above.

TFIZ1 was expressed by mucus secreting epithelial cells along the mucosal surface and in the foveolae throughout the stomach in all cases examined (Fig. 2). It was not detected in the neck region or basal portion of gastric glands or in the submucosa. The expression profile of TFF1 coincided with that of TFIZ1. TFF2 was expressed in mucus neck cells and in cells in the basal portion of gastric glands. TFF3 was not detected in normal gastric mucosal cells in this study. TFIZ1, like TFF1, was detected in the cytoplasm and in mucus globules in the apical region of the secretory cells (Fig. 2). There was a diffuse immuno-reaction in the cytoplasm towards the basolateral surface of the cells and a more intense immuno-reaction in mucus globules towards the apical surface. These results, therefore, demonstrate consistent co-localisation of TFIZ1 and TFF1 in specific mucus secreting epithelial cells of the normal human stomach.

### 3.3 Interaction of TFIZ1 with the trefoil proteins TFF2 and TFF3

**Fig. 3:**
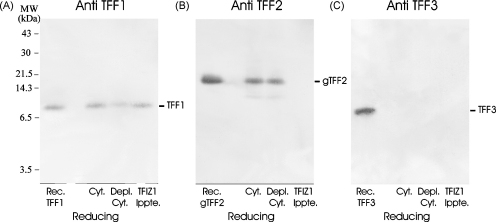
Investigation of interaction between TFIZ1 and TFF1, TFF2 or TFF3 in human gastric mucosa. TFIZ1 was immunoprecipitated from gastric cytosol. Aliquots of recombinant TFF1, glycosylated TFF2 (gTFF2) or TFF3 and of gastric cytosol (Cyt.), immuno-depleted cytosol (Depl. Cyt.) and TFIZ1 immunoprecipitate (TFIZ1 Ippte.) were electrophoresed on polyacrylamide gels after incubation with β-mercaptoethanol. The proteins were transferred to PVDF membrane and incubated with TFF1 antibody (A), TFF2 antibody (B) or TFF3 antibody (C). The positions of the molecular mass markers are shown on the left and of the trefoil proteins on the right of the panels.

The possibility that TFIZ1 forms heterodimers *in vivo* with the other two human trefoil proteins was pursued in further experiments for two reasons. The first was that the murine orthologue of TFIZ1 was identified recently in an *in vitro* experiment, ligand blot analysis, as the molecular partner of TFF2 even though TFF2 does not contain a free cysteine residue. The second was that TFF3, like TFF1, contains an unpaired cyteine residue close to its carboxy-terminus and can also form homodimers. TFIZ1 was immunoprecipitated from gastric cytosol under non-denaturing conditions, and analysed with TFF1, TFF2 and TFF3 antibodies after electrophoresis under reducing conditions and Western transfer (Fig. 3). TFF1 was co-immunoprecipitated by the TFIZ1 antibody (Fig. 3A). In contrast, TFF2 and TFF3 were not. TFF2 was detected readily in whole and immuno-depleted gastric cytosol but was not detected in the TFIZ1 immunoprecipitate (Fig. 3B). In the reciprocal experiment in which TFF2 was immunoprecipitated from human gastric cytosol with a specific TFF2 monoclonal antibody, no TFIZ1 was detected in the immunoprecipitate (data not shown). TFF3 was not detected in gastric cytosol or in the proteins immunoprecipitated by TFIZ1 antiserum (Fig. 3).

### 3.4 Specificity of dimer formation between TFIZ1 and TFF1

Both TFF1 and TFIZ1 contain an odd number of cysteine residues and each, therefore, contains one free cysteine which is potentially able to form homo- or heterodimers. Immunoprecipitation experiments were used to determine the major molecular forms of each protein and to examine the promiscuity of TFIZ1 to form heterodimers with non-trefoil proteins in gastric mucosa.

**Fig. 4:**
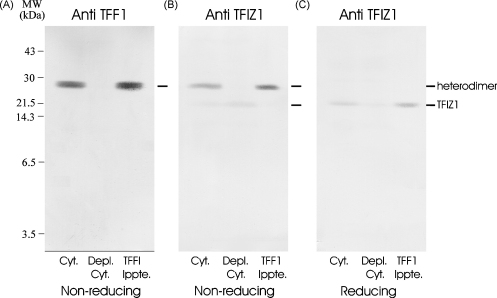
TFIZ1 is present as a monomer and a heterodimer in human gastric mucosa. TFF1 was immunoprecipitated from gastric cytosol. Aliquots of gastric cytosol (Cyt.), immuno-depleted cytosol (Depl. Cyt.) and TFF1 immunoprecipitate (TFF1 Ippte.) were electrophoresed on polyacrylamide gels without (A and B) or after (C) prior incubation with β-mercaptoethanol. The proteins were transferred to PVDF membrane and incubated with TFF1 antibody (A) or TFIZ1 antiserum (B and C). The positions of the molecular mass markers are shown on the left and of the different forms of TFIZ1 on the right of the panels.

Aliquots of cytosol from normal human gastric mucosa, TFF1 immuno-depleted cytosol and proteins immunoprecipitated by TFF1 antibody were analysed by Western transfer analysis under non-reducing (Fig. 4A and B) and reducing (Fig. 4C) conditions. The major TFF1 immuno-reactive protein band under non-reducing conditions is the heterodimer with TFIZ1 and all was removed by immunoprecipitation (Fig. 4A). TFIZ1 antisera detected two protein bands (Fig. 4B) in gastric cytosol. The antibody reacts strongly with a protein band of ∼25 kDa that co-migrates with the TFF1:TFIZ1 heterodimer and more weakly with a more rapidly migrating protein band. The TFIZ1 antibody reacts only with the more rapidly migrating band in the TFF1 immuno-depleted cytosol and only with the ∼25 kDa protein band in the TFF1 immunoprecipitate. These results are consistent with TFIZ1 being present in at least two molecular forms in normal human mucosa: as a heterodimer with TFF1 that is immunoprecipitated by TFF1 specific antibodies and as a TFIZ1 monomer that is not immunoprecipitated by TFF1 antibody. No other molecular forms were detected suggesting that TFIZ1 does not readily form heterodimers with other proteins nor does it have a propensity to form homodimers. Western transfer analysis of the same samples under reducing conditions with TFIZ1 antibody (Fig. 4C) revealed a single protein, that migrates more slowly than would be predicted from its theoretical molecular mass of 18.3 kDa as observed previously (Westley et al., 2005).

**Fig. 5:**
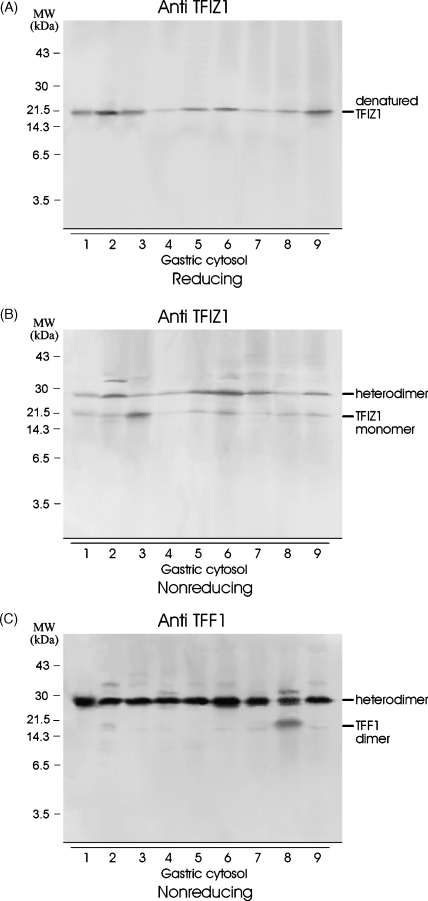
Expression of different molecular forms of TFIZ1 and TFF1 in normal gastrointestinal mucosa. Aliquots of cytosol prepared from gastric mucosa were electrophoresed on a polyacrylamide gel with (A) or without (B and C) prior incubation with β-mercaptoethanol. The separated proteins were transferred to PVDF membrane and incubated with TFIZ1 antiserum (A and B) or TFF1 antibody (C). The positions of the molecular mass markers are shown on the left and of the different forms of the proteins on the right of the panels.

To examine the variability of TFIZ1 and TFF1 expression between individuals, gastric mucosal extracts of nine individuals were analysed by Western transfer analysis. TFIZ1 was detected in variable amounts in all nine samples analysed (Fig. 5A). Highest levels of TFIZ1 were detected in cytosols 1, 2, 3, 5, 6 and 9 and lowest levels in 4 and 7. Analysis under non-reducing conditions indicated firstly that the TFF1:TFIZ1 heterodimer was present in all samples but the amount differed and secondly that the ratio of TFIZ1 in the monomeric form and the TFF1:TFIZ1 heterodimeric form varied. For example, the majority of TFIZ1 is in the monomeric form in cytosol 3 whereas the majority is in the heterodimeric form in cytosol 7 (Fig. 5B). Analysis with the TFF1 antibody under non-reducing conditions confirmed that the TFF1:TFIZ1 heterodimer is present in all samples (Fig. 5C). The heterodimer is the predominant form of TFF1, but in cytosol 8 which has relatively high concentrations of TFF1 compared to TFIZ1, the TFF1 homodimer was readily detected. This suggests that the relative amounts of the different molecular forms of TFF1 and TFIZ1 are determined in part by the relative concentrations of the two proteins so that if the concentration of TFIZ1 becomes limiting, TFF1 is more likely to form homodimers. This experiment found no strong evidence for heterodimerisation of TFF1 or TFIZ1 with other proteins.

### 3.5 Expression of TFIZ1 and TFF1 in human gastric tumours

**Fig. 6:**
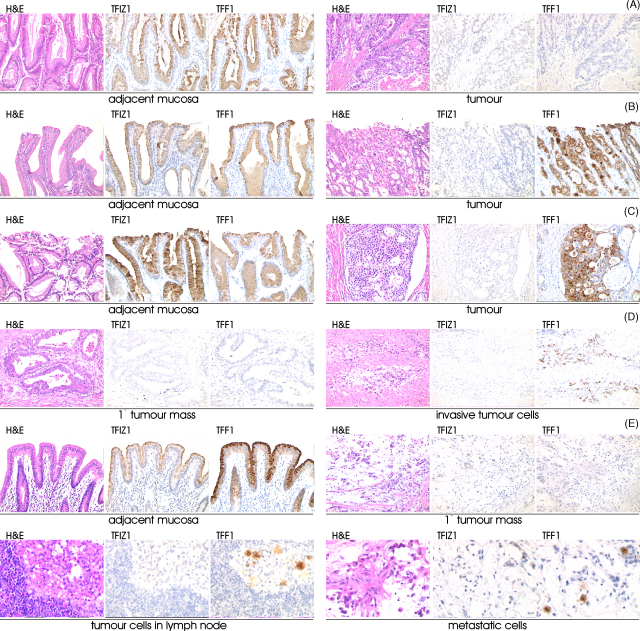
Loss of expression of TFIZ1 and TFF1 in human gastric adenocarcinoma. Sections of non-involved gastric mucosa, primary gastric tumour mass and of metastatic carcinoma cells were processed for immunohistochemistry to locate TFIZ1 and TFF1. Sections are shown stained with haematoxylin and eosin (H&E) to allow visualization of the cellular structures. Sections are from a small well differentiated tumour (A), a small well to moderately differentiated tumour (B), a 1.5 cm × 2.5 cm moderately to poorly differentiated tumour (C), a 3.5 cm × 5.0 cm poorly differentiated tumour with local infiltration (D) and a 3.0 cm × 5.0 cm poorly differentiated tumour with metastatic deposits (E). The photomicrograph of the 1.5 cm × 1.5 cm well to moderately differentiated tumour shows well differentiated tumour cells on the right and moderately differentiated tumour cells to the left (B). The original magnifications for the photomicrographs is ×200.

TFF1 is a gastric specific tumour suppressor. The protein to which it is bound in the normal stomach may impact on its behaviour in gastric cancer. We investigated co-expression of TFIZ1 and TFF1 in a series of 15 primary gastric tumours and corresponding metastatic tumour cells (Table 1). Both proteins were co-expressed in non-neoplastic tissue adjacent to the primary tumours (Fig. 6). In seven of the cases examined, neither TFIZ1 nor TFF1 were detected in the tumour cells. Expression was lost even in very early gastric cancers. For instance, in the case shown in Fig. 6A, TFIZ1 and TFF1 were detected readily in non-involved mucosa but were not detected in carcinoma cells. In eight tumours, expression of TFF1 was detected in the absence of TFIZ1. For instance for a small tumour (Fig. 6B), TFIZ1 expression was detected only in the non-involved mucosa whereas TFF1 was detected in non-involved mucosa and in both well and moderately differentiated carcinoma cells. Similarly in a moderately to poorly differentiated tumour (Fig. 6C), TFIZ1 expression was detected only in adjacent mucosa, whereas TFF1 was detected in almost all carcinoma cells. Loss of or weak TFF1 expression in the main tumour mass was contrasted frequently with strong expression of TFF1 but not TFIZ1 by carcinoma cells at the infiltrating edge or disseminated through the submucosa. In an extreme example, TFF1 expression was absent in the main tumour mass but was present in tumour cells that had infiltrated the stroma (Fig. 6D). TFIZ1 expression was not detectable within the infiltrating tumour cells that express TFF1 or in any other tumour cells. In a second example, TFIZ1 and TFF1 were detected in adjacent normal mucosa, neither was detected in the primary tumour, but TFF1 was expressed by metastatic carcinoma cells located within a lymph node from the greater curve and by metastatic carcinoma cells that have invaded fat from the right paracardial area (Fig. 6E).

The expression of TFF1 in the absence of TFIZ1 in infiltrating and metastatic tumour cells suggested that this pattern of expression might be associated with lymph node involvement. Metastatic carcinoma cells were not present in lymph nodes in five of the seven cases in which neither protein was detected. In all eight cases, in which TFF1 was detected in the absence of TFIZ1, metastatic carcinoma cells had disseminated to lymph nodes (Table 1). The data showed that expression of TFF1 in the absence of TFIZ1 was associated with lymph node involvement (Fisher's exact; *p* = 0.007) and suggested that it predisposes towards tumour spread.

## 4 Discussion

The TFF1:TFIZ1 heterodimer is the major form of TFF1 in normal human gastric mucosa (Ruchaud-Sparagano et al., 2004; Newton et al., 2000; Westley et al., 2005). In this study, we show that the heterodimer is also the major form of TFIZ1 which suggests that this covalent protein–protein interaction is specific for these two proteins and that neither protein is significantly promiscuous in its ability to form heterodimers with other proteins and that the formation of the TFF1:TFIZ1 heterodimer is critical for the normal biological function of both proteins. The relative proportions of both TFIZ1 and TFF1 monomer and heterodimer varied somewhat between individuals. Interestingly, the proportions of the molecular forms of TFF1 and TFIZ1 can be affected by the relative abundance of the other protein. For instance, if less TFIZ1 is expressed, more of the TFF1 dimer is formed. This may have biological implications as it is known that the TFF1 dimer stimulates the migration of tumour cells (Prest et al., 2002).

The demonstration that TFF1, but not the other structurally related trefoil proteins, TFF2 or TFF3, is co-immunoprecipitated by TFIZ1 antiserum combined with the co-immunoprecipitation of the majority of TFIZ1 from gastric cytosol by a TFF1 monoclonal antibody and the inability of a TFF2 antibody to co-immunoprecipitate TFIZ1 with TFF2 suggest that in normal human gastric cells TFIZ1 reacts specifically with TFF1 and not with TFF2 or TFF3. In the case of TFF2, TFIZ1 and TFF2 are synthesized in different cell types and a heterodimer, if formed, would probably not be stabilised by a disulphide bond because TFF2 has no unpaired cysteine residue. The murine orthologue of TFIZ1 was identified recently by ligand blot analysis with a fusion protein of murine TFF2 which suggested that TFIZ1 interacts with TFF2 (Otto et al., 2006). Our results are not consistent with this as TFF2 and TFIZ1 are not co-localised in the gastric mucosa and there was no evidence for a TFF2:TFIZ1 heterodimer from co-immunoprecipitation experiments. In the case of TFF3, which does contain a free cysteine residue in a similar position to TFF1, a TFF3:TFIZ1 heterodimer is unlikely to be formed because TFF3 is absent from or expressed at low levels in the normal stomach. There are also significant structural differences between TFF1 and TFF3 which may preclude the formation of a TFF3:TFIZ1 heterodimer (May et al., 2003).

TFIZ1 protein expression in the gastric mucosa is restricted to cells that express TFF1. TFIZ1 is co-localised with TFF1 in mucus secretory cells on the surface and in the foveolae throughout the normal human stomach. The strongest immuno-reaction for both TFIZ1 and TFF1 is within the mucus secretory vesicles which is compatible with our demonstration that the TFF1:TFIZ1 heterodimer is present in adherent gastric mucus (Newton et al., 2000).

This is the first study of TFIZ1 protein expression in gastric cancer and demonstrates that TFIZ1 expression is lost in gastric cancer even in very early tumours. This is consistent with an early report in the Chinese literature that the expression of the mRNA that encodes the TFIZ1 protein is reduced in gastric cancer (Du et al., 2003). TFF1 protein expression is also lost in gastric tumours, but in about half the cases in which TFIZ1 protein was not expressed, TFF1 expression was detected. In some cases, in which expression of both proteins was absent in the primary tumour, TFF1 but not TFIZ1 was expressed in infiltrating and metastatic tumour cells. The finding that TFF1 is expressed in the absence of TFIZ1 in some gastric tumours and further that an ability to express TFF1 but not TFIZ1 is acquired by invasive cancer cells suggest that this phenotype might be associated with lymph node metastasis and poor prognosis. Our demonstration of an association between TFF1 expression in the absence of TFIZ1 expression and lymph node involvement supports this contention.

Work with TFF1-null mice showed that TFF1 is a gastric tumour suppressor (Lefebvre et al., 1996). The gastric mucosa of TFF1-null mice becomes thickened by three weeks of age and by five months exhibits severe hyperplasia, dysplasia and absence of mucin. All mice develop antropyloric adenomas and 30% develop adenocarcinomas. Loss of TFF1 expression occurs frequently in human gastric cancer (Henry et al., 1991; Muller and Borchard, 1993). TFF1 gene inactivation occurs by loss of heterozygosity and hypermethylation of the TFF1 promoter (Park et al., 2000b; Fujimoto et al., 2000; Carvalho et al., 2002). Missense mutations in TFF1 have been found in gastric cancers however their importance is uncertain (Carvalho et al., 2002; Park et al., 2000a; Beckler et al., 2003).

In seeming contradiction to its proposed role as a tumour suppressor, TFF1 is frequently over-expressed or expressed ectopically by tumour cells (May and Westley, 1997). It has been suggested that TFF1 may be involved in tumour dissemination because it stimulates migration and invasion of tumour cells (Prest et al., 2002; Emami et al., 2001). Inappropriate TFF1 expression may also promote tumourigenesis due to its anti-apoptotic and angiogenic activities (Bossenmeyer-Pourie et al., 2002; Rodrigues et al., 2003). TFF1 expression is associated with increased incidence of breast cancer metastasis to bone (Smid et al., 2006), high levels of TFF1 expression in metastatic tumour cells renders it a reliable marker of micrometastases (Mikhitarian et al., 2005) and expression is increased in tumours that arise during the interval between routine breast cancer screening (Crosier et al., 2001).

The dissociation of TFF1 and TFIZ1 protein expression in gastric carcinoma cells could provide an explanation for the apparent contradiction that TFF1 expression is beneficial in normal tissue but detrimental in cancerous tissue (May and Westley, 1997). It may also reconcile the observation that a gastric tumour suppressor is expressed ectopically by other tumours and at high levels by a large proportion of breast tumours including interval cancers (Henry et al., 1991; Crosier et al., 2001; Rio et al., 1987; Foekens et al., 1994). Our results suggest that expression of TFF1 in the absence of the interacting protein TFIZ1 confers an invasive phenotype on tumour cells and that TFIZ1 may itself be a tumour suppressor or that it is the formation of the heterodimer that is important for the tumour suppressor activity of TFF1. The consistent, early and permanent loss of TFIZ1 expression in human gastric tumours is supportive of these possibilities. Clearly, absence of TFIZ1 will affect the molecular forms of TFF1 produced and may favour production of the TFF1 dimer which stimulates cancer cell motility and invasion. Our data suggest that expression of TFIZ1 might ensure normal, beneficial function of TFF1 whereas in the absence of TFIZ1, TFF1 promotes invasion.

In a recent expression microarray analysis of the effects of *H. pylori* eradication on gene expression in gastric mucosal surface epithelial cells, TFIZ1 mRNA was the most induced mRNA which indicates that TFIZ1 expression is reduced following *H. pylori* infection (Resnick et al., 2006). If TFIZ1 has tumour suppressor activity, reduction of TFIZ1 expression after *H. pylori* infection may explain in part the carcinogenic role of *H. pylori* in the progression to gastric cancer. In addition, continued expression of TFF1 in the context of loss of TFIZ1 expression induced by *H. pylori* infection could facilitate prolonged colonization of the mucus gel layer by *H. pylori* (Clyne et al., 2004). In the longer term, persistence of the imbalance in TFIZ1 and TFF1 expression could increase the invasive behaviour of any tumour induced.

## Figures and Tables

**Table 1:** Characteristics of gastric tumours analysed by immunohistochemistry

Tumour	Tumour size (cm)	Differentiation	Tumour stage	Nodal status
1	6.0 × 5.0	Moderate	T3	Positive
2	5.0 × 5.5	Poor	T4	Positive
3	1.8 × 1.2	Well	T1	Negative
4	2.5 × 1.5	Moderate–poor	T3	Negative
5	5.5 × 3.5	Poor	T3	Negative
6	1.8 × 1.8	Moderate–poor	T2	Negative
7	3.5 × 5.0	Poor	T3	Positive
8	4.5 × 4.5	Moderate	T2	Positive
9	5.3 × 5.2	Poor	T2	Negative
10	2.5 × 4.0	Well-moderate	T2	Negative
11	<2 × <1.0	Well	T1	Negative
12	4.0 × 3.5	Poor	T3	Positive
13	1.5 × 1.5	Moderate	T2	Positive
14	3.0 × 5.0	Poor	T3	Positive
15	4.2 × 3.0	Moderate–poor	T3	Positive
